# Regulation of Cell Cycle Regulators by SIRT1 Contributes to Resveratrol-Mediated Prevention of Pulmonary Arterial Hypertension

**DOI:** 10.1155/2015/762349

**Published:** 2015-07-26

**Authors:** Shuang Zhou, Meng-Tao Li, Yu-Yan Jia, Jin-Jing Liu, Qian Wang, Zhuang Tian, Yong-Tai Liu, Hou-Zao Chen, De-Pei Liu, Xiao-Feng Zeng

**Affiliations:** ^1^Department of Rheumatology, Peking Union Medical College Hospital, Beijing 100032, China; ^2^State Key Laboratory of Medical Molecular Biology, Department of Biochemistry and Molecular Biology, Institute of Basic Medical Sciences, Chinese Academy of Medical Sciences & Peking Union Medical College, Beijing 100005, China; ^3^Department of Cardiology, Peking Union Medical College Hospital, Beijing 100730, China

## Abstract

Pulmonary arterial hypertension (PAH) is a major cause of morbidity and mortality in rheumatic diseases. Vascular remodeling due to the proliferation of pulmonary arterial smooth muscle cells (PASMCs) is central to the development of PAH. To date, it is still unclear if Silence Information Regulator 1 (SIRT1) regulates cell cycle regulators in the proliferation of PASMCs and contributes to prevention of PAH by resveratrol. In this study, we found that a significant decrease of SIRT1 expression levels in platelet-derived growth factor BB (PDGF-BB) treated human PASMCs (HPASMCs) and in monocrotaline (MCT) induced PAH rat. Overexpression of SIRT1 induced G1 phase arrest and increased p21 expression but decreased cyclin D1 expression in PDGF-BB treated HPASMCs. Moreover, resveratrol attenuated pulmonary arterial remodeling, decreased pulmonary arterial pressure, and upregulated SIRT1 and p21 expression but downregulated cyclin D1 expression in MCT induced PAH rat. Notably, knockdown of SIRT1 eliminated the regulation of resveratrol on p21 and cyclin D1 expression in PDGF-BB treated HPASMCs. These results demonstrated that SIRT1 mediated the regulation of resveratrol on the expression of cell cycle regulatory molecules. It suggests that SIRT1 exerts a protective role in PAH associated with rheumatic diseases and can be a potential treatment target.

## 1. Introduction

Pulmonary arterial hypertension (PAH) is a progressive disease characterized by an increase in pulmonary vascular resistance leading to right ventricular (RV) failure and death [1]. PAH can occur in a variety of other conditions and circumstances including a number of rheumatic diseases. In this regard, systemic sclerosis (SSc), mixed connective tissue disease (MCTD), and systemic lupus erythematodes (SLE) are associated with PAH [2]. In community-based rheumatology practices in the US, the prevalence of PAH was 13.3% in patients with SSc and MCTD as analysed by echocardiography [3]. PAH is a major cause of morbidity and mortality in connective tissue diseases. While 3-year survival rates after diagnosis in idiopathic PAH (IPAH) are as low as 48%, these alarming numbers are even worse in PAH associated with SSc [2]. This survival rate highlights the need for early diagnosis and treatment of PAH associated with rheumatic diseases [2].

Despite its heterogeneous origin, it is generally accepted that the pathogenesis of PAH involves three major processes including vasoconstriction, vascular remodeling characterized by enhanced proliferation of pulmonary arterial smooth muscle cells (PASMCs) and endothelial cells and coagulation abnormalities [4]. The traditional methods such as treatment with calcium-channel blockers and anticoagulants have limited function, while current therapies such as endothelin receptor antagonists [5, 6], phosphodiesterase 5 inhibitors [7–10], and prostacyclin analogs [11–13] are developed mainly as vasodilators. Although these therapies appear to somewhat improve the quality of life of PAH patients, the prognosis remains poor [14]. Novel treatments are required to prevent progression of pulmonary hypertension by interfering with the pathomechanisms of the disease at multiple levels [15]. For example, in a preclinical setting, experimental therapeutics that exert antimitogenic effects on proliferation of PASMCs [16–18], addition to promoting vasodilation, show promise in enhancing overall prognosis.

Resveratrol (3,5,4-trihydroxystilbene) is a dietary polyphenolic compound that exerts significant antioxidant, anti-inflammatory, and endothelial protective effects in the systemic circulation [19–22]. Resveratrol prevents MCT induced pulmonary hypertension in rats [15]. Resveratrol, a Silence Information Regulator 1 (SIRT1) activator, induces apoptosis MH7A human rheumatoid arthritis synovial cells in a SIRT1-dependent manner [23]. SIRT1, a NAD-dependent histone deacetylase, has been implicated in aging, metabolism, and tolerance to oxidative stress via its ability to deacetylate a variety of substrates, including histones, transcription factors, and coregulators [24]. SIRT1 attracts lots of interest in its cardiovascular protective role, which serves as a key regulator in vascular endothelial homeostasis by controlling angiogenesis, vascular tone, and endothelial dysfunction as well as by decreasing atherosclerosis [25–28]. Our previous study has shown that SIRT1 overexpression markedly inhibited vascular smooth muscle cell (VSMC) proliferation and migration and induced cell cycle arrest at G1/S transition* in vitro* [29]. Whether SIRT1 mediates the protective role of resveratrol on PAH and the mechanism that SIRT1 inhibits HPASMCs proliferation is still unknown. We therefore hypothesize that SIRT1 may inhibit HPASMCs proliferation through affecting the cell cycle regulator and contribute to prevention of PAH by resveratrol.

In this study, we found that SIRT1 regulated the expression of cell cycle regulatory molecules and arrested PDGF-BB-treated HPASMCs in G0/G1 phase. Decreased SIRT1 expression was found in an experimental rat PAH model induced by MCT. Resveratrol attenuated MCT induced rat pulmonary arterial remodeling and decreased pulmonary arterial pressure. Meanwhile, resveratrol increased SIRT1 and p21 expressions but decreased cyclin D1 expression in PAH rat model. Furthermore, resveratrol regulated the expression of cell cycle regulatory molecules in HPASMCs in a SIRT1-dependent manner.

## 2. Materials and Methods

### 2.1. Drugs

Recombinant human PDGF-BB was purchased from R&D systems. Sterile 4 mmol/L HCL containing 0.1% BSA was added to the vial to prepare a stock solution of 100 *μ*g/mL of PDGF-BB. Resveratrol was purchased from Sigma.

### 2.2. Animals

All of the animal protocols were approved by the Animal Care and Use Committee at the Institute of Basic Medical Sciences, Chinese Academy of Medical Sciences & Peking Union Medical College in accordance with the Guide for the Care and Use of Laboratory Animals published by the US National Institutes of Health (NIH Publication, 8th Edition, 2011). Adult male Sprague-Dawley rats (280–300 g in body weight) were randomly assigned to the following groups (*n* = 20 animals in each group): (1) control animals (receiving a s.c. injection of saline) receiving vehicle treatment (saline, given orally) for 14 or 21 days; (2) MCT (receiving a s.c. injection of MCT with a dose of 60 mg·kg^−1^) challenged animals receiving vehicle treatment (saline, given orally) for 14 or 21 days; (3) MCT challenged animals receiving resveratrol (2.5 mg·kg^−1^d^−1^, given orally) from day 1 to day 14 or day 21 after MCT injection; (4) MCT challenged animals receiving resveratrol (20 mg·kg^−1^d^−1^, given orally) from day 1 to day 14 or day 21 after MCT injection.

### 2.3. Assessment of Hemodynamics and Right Ventricle (RV) Hypertrophy

The animals were anesthetized with ketamine (60 mg·kg^−1^ IM) and xylazine (3 mg·kg^−1^ IM). Efficiency of anaesthesia was monitored by lack of withdrawal reflex upon hind toe pinching, and no reaction to skin pinch over the area to be incised. A right heart catheter was inserted into the right jugular vein then pushed through the right ventricle (RV) into the pulmonary artery for measurement of mean pulmonary arterial pressure (mPAP) and RV systolic pressure (RVSP) [30]. Another polyethylene catheter was inserted into the left carotid artery to measure the systemic arterial pressure (SAP). The heart was dissected and weighed. The right ventricular hypertrophy index (RVHI) was calculated as the ratio of RV free wall weight divided by the sum of the free left ventricle (LV) wall and ventricular septum [17].

### 2.4. Immunohistology

After measurement of hemodynamics, rats were perfused with phosphate-buffered saline and the thorax was opened and the left lung immediately removed and fixed in the distended state with formalin buffer. The media thickness was measured in Verhoeff Van Gieson (EVG) sections (5 *μ*m thick). EVG staining was performed using a EVG staining kit (Baso) according to the manufacturer's instructions. 5-6 rats were used in each group and in each rat, 10 microscopic fields were chosen at random. The media thickness was expressed as the following formula: index% = (external diameter − internal diameter)/external diameter. The internal and external diameter were calculated according to the lamina elastic internal and the lamina elastic external indicated by EVG staining in a blinded manner by a single observer using Image Pro Plus 6.0 software (Media Cybernetics) [31]. According to the external diameter, arterials were categorized to three groups as diameter between 25–50 *μ*m, 51–100 *μ*m, and 101–500 *μ*m. The muscularization degrees of pulmonary arteries were measured in *α*-smooth muscle actin (*α*-SMA) staining sections. For the analysis of pulmonary arteries muscularization degrees, slides were deparaffinized, and endogenous peroxidase activity was quenched with 3% hydrogen peroxide in 1 × PBS for 10 minutes. Nonspecific binding sites were blocked in 10% goat serum in PBS at room temperature for 1 hour. Slides were incubated at 4°C overnight with anti-*α*-smooth muscle actin rabbit polyclonal antibody (Abcam) and then with biotinylated secondary antibody at 37°C for 30 minutes and subsequently with horseradish peroxidase-labeled streptavidin solution for 20 minutes at 37°C. Slides were then stained with diaminobenzidine and counterstained with hematoxylin. In each rat, 10 microscopic fields were chosen at random and a total of about 80 intraacinar arteries were categorized as muscular (i.e., with a complete medial coat of muscle), partially muscular (i.e., with only a crescent of muscle), or nonmuscular (i.e., no apparent muscle), as previously reported [17].

### 2.5. PASMCs Apoptosis Assessment

Apoptosis of rat PASMCs were evaluated with the TdT-mediated dUTP nick and labeling (TUNEL) assay. The TUNEL assay was performed using a DeadEnd Fluorometric TUNEL System (Promega) according to the manufacturer's instructions. Briefly, 5 *μ*m-thick tissue sections were obtained from the samples. The slides were washed with 1 mL of the wash buffer provided in the kit and labeled with the DNA labeling solution at 37°C for 60 minutes. After the slides were rinsed and incubated with equilibration buffer, nucleotide mix, and rTdT enzyme at 37°C for 1 hour in the dark, they were counterstained with Hoechst and then examined using confocal fluorescence microscopy. TUNEL positive cells were identified by a green fluorescence.

### 2.6. Flow Cytometry Analysis

Adenovirus-infected HPASMCs were maintained in serum-free M231 medium for 24 hours and then stimulated with 10 ng/mL PDGF-BB for different periods. The cells were trypsinized, fixed in 70% ethanol at 4°C overnight, washed twice with ice-cold PBS, and incubated with RNase and propidium iodide. The cell-cycle phase was analyzed by flow cytometry using a Becton Dickinson FACStar flow cytometer and the Becton Dickinson CellFIT software.

### 2.7. Cell Culture, Adenovirus Generation, and Infection

HPASMCs (Invitrogen) were cultured in M231 medium (Invitrogen) with smooth muscle growth supplement. The replication-defective adenoviral vectors expressing SIRT1 (Ad-SIRT1) or control green fluorescent protein (Ad-GFP) and adenovirus-mediated knockdown of SIRT1 (Ad-SIRT1 RNAi) or control vector (Ad-U6) were generated as described previously [26, 32]. HPASMCs were infected for 2 hours with the above adenovirus using a multiplicity of infection (MOI) of 100, washed, and incubated in serum-free medium without virus for at least 24 hours before drugs challenge.

### 2.8. Reverse Transcription and Real-Time PCR

Total RNA was extracted from cells or lungs of rats using Trizol (Invitrogen) according to the manufacturer's instructions. Two micrograms of total RNA were used to synthesize first-strand cDNA with M-MuLV reverse transcriptase (New England BioLabs) using random primers. Real-time PCR was performed using the BioRad iCycler iQ5 Real-Time PCR Detection System with the Quantitect SYBR Green One-Step RT-PCR Kit (QIAGEN). Fluorescence curves were analyzed with iCycler iQ5 Optical System Software (Version 2.0).

### 2.9. Western Blotting

Cellular and rat tissue proteins were extracted using RIPA buffer (25 mM Tris–HCl pH 7.6, 150 mmol/L NaCl, 1% NP-40, 1% sodium deoxycholate, and 0.1% SDS). After complete homogenization on an ice rotator, samples were sonicated and centrifuged at 4°C. The supernatants were transferred into fresh tubes and protein concentrations were determined by the BCA method. Equal amounts of protein (20 *µ*g/lane) were separated by SDS-PAGE and transferred onto polyvinylidene difluoride membranes (Millipore). After being blocked, the filters were incubated with the following primary antibodies: anti-SIRT1 (Santa Cruz Biotechnology), anti-p21 (Santa Cruz Biotechnology), anti-cyclin D1 (Santa Cruz Biotechnology), anticyclin E (Santa Cruz Biotechnology), anti-GAPDH (Santa Cruz Biotechnology), anti-CDK2 (Santa Cruz Biotechnology), and anti-CDK4 (Santa Cruz Biotechnology). After being washed and incubated with the appropriate horseradish peroxidase-conjugated secondary antibody (Santa Cruz Biotechnology), the immune complexes were visualized with a chemiluminescence reagent. Western blots were quantified densitometrically with Quantity One software (Bio-Rad), and the intensity values were normalized to GAPDH.

### 2.10. Statistics

Data are expressed as means ± SEM. Statistical analyses were performed by a two-tailed unpaired student's *t*-test or a one-way ANOVA as appropriate to determine statistical significance between the groups. A *P* value less than 0.05 was considered significant.

## 3. Results

### 3.1. PDGF-BB Affects Cell Cycle Regulatory Molecules and SIRT1 Expression

**Figure 1:**
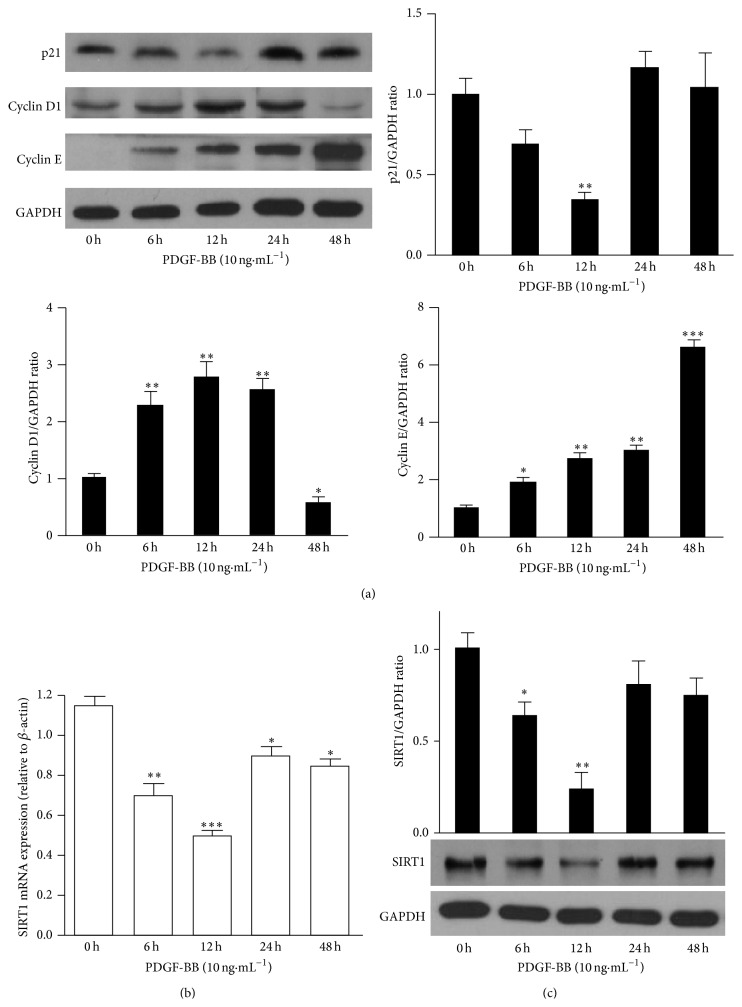
PDGF-BB affects cell cycle regulatory molecules and SIRT1 expressions. After 24-hour serum starvation, HPASMCs were treated with 10 ng·mL^−1^ PDGF-BB and harvested at the indicated time points. p21, cyclin D1, cyclin E (a), and SIRT1 (c) protein levels were analyzed by western blotting. Bar graphs show densitometric analysis of western blotting. The densitometric quantification was normalized to GAPDH. Data are shown as means ± SEM for three independent experiments. SIRT1 mRNA level was analyzed by real-time PCR (b) (*n* = 3). mRNA level was normalized to the internal control *β*-actin. ^*^ *P* < 0.05, ^**^ *P* < 0.01, ^***^ *P* < 0.001 versus 0 hour.

Vascular remodeling is a critical step of PAH progression and is characterized by proliferation of PASMCs. The development of vascular remodeling requires cells arrested in the G0/G1 phase to enter the cell cycle [33]. p21, cyclin D1, and cyclin E are key regulators in the switch of G0/G1 phase to S phase and in VSMC proliferation [33–35]. PDGF-BB, a potent mitogen involving in proliferation and migration of PASMCs, has been proposed as a key mediator in the progression of PAH [17]. PDGF-BB (10 ng·mL^−1^) treatment decreased expression of p21 but increased cyclin D1 and cyclin E expression in HPASMCs (Figure 1(a)), while the expression of CDK2 and CDK4 made no remarkable change (see Figure S1 in Supplementary Material available online at http://dx.doi.org/10.1155/2015/762349). We observed that PDGF-BB treatments significantly decreased SIRT1 mRNA (Figure 1(b)) and protein (Figure 1(c)) expression in HPASMCs.

### 3.2. SIRT1 Regulates Expression of Cell Cycle Regulatory Molecules and Arrests PDGF-BB-Treated HPASMCs in G0/G1 Phase

**Figure 2:**
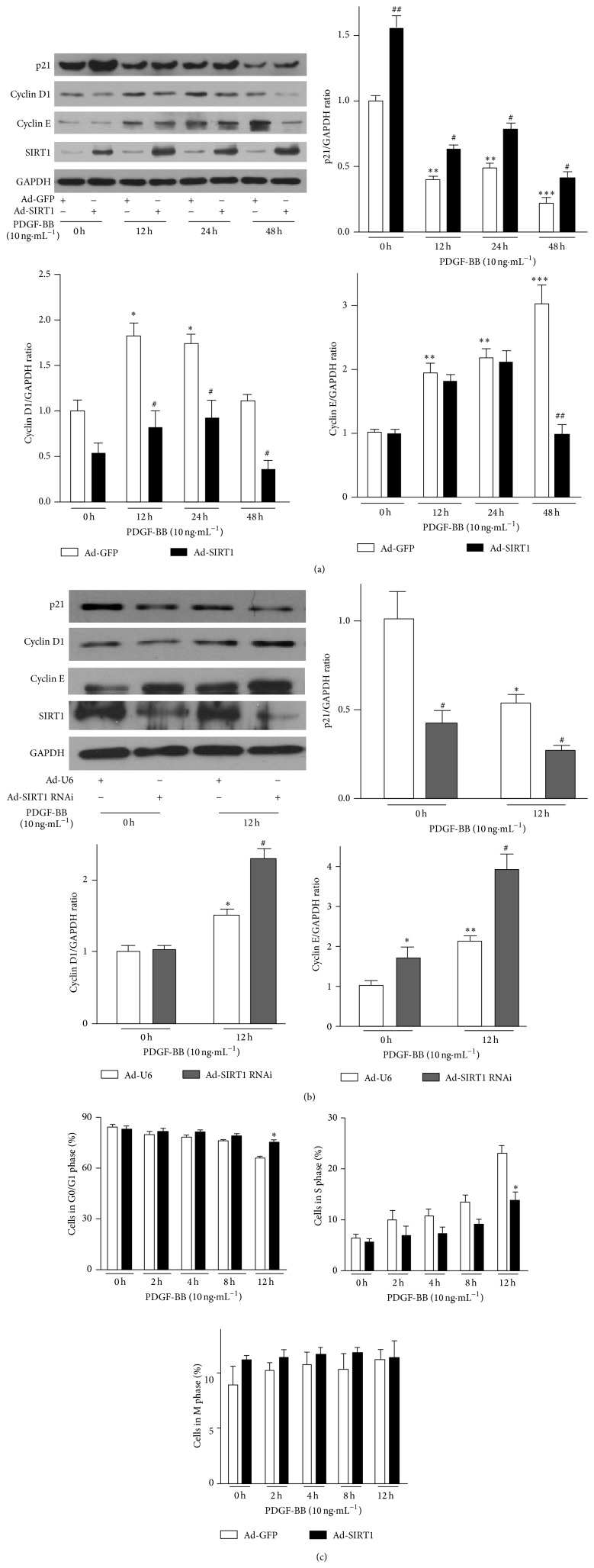
SIRT1 regulates expression of cell cycle regulatory molecules and arrests PDGF-BB-treated HPASMCs in G0/G1 phase. HPASMCs infected with adenoviral vectors encoding SIRT1 overexpression (a) or knockdown (b) were treated with PDGF-BB for indicated period. p21, cyclin D1, and cyclin E protein levels were analyzed by western blotting. Bar graphs show densitometric analysis of western blotting. The densitometric quantification was normalized to GAPDH. Data are shown as means ± SEM for three independent experiments. ^*^ *P* < 0.05, ^**^ *P* < 0.01, and ^***^ *P* < 0.001 versus 0 hour Ad-GFP group (a) or 0 hour Ad-U6 group (b); ^#^ *P* < 0.05, ^##^ *P* < 0.01 versus corresponding Ad-GFP group (a) or Ad-U6 group (b) at the indicated time points. (c) Flow cytometry analysis of HPASMCs after SIRT1 overexpression and treatment with PDGF-BB (*n* = 3). ^*^ *P* < 0.05 versus Ad-GFP at the indicated time points.

SIRT1 overexpression significantly increased p21 expression, however, decreased cyclin D1 and cyclin E expression in PDGF-BB treated HPAMSCs. (Figure 2(a)). Overexpression of SIRT1 decreased CDK2 expression in PDGF-BB treated HPAMSCs but had no effect on CDK4 expression (Figure S2A). Knockdown of SIRT1 showed a marked decrease in p21 expression and an increase in cyclin D1, cyclin E (Figure 2(b)), and CDK2 expression levels (Figure S2B) in PDGF-BB treated HPAMSCs. However, CDK4 expression remained unchanged (Figure S2B). Flow cytometry analysis showed significant G0/G1 accumulation of HPASMCs with SIRT1 overexpression 12 hours after PDGF-BB treatment (Figure 2(c)). These results indicated that SIRT1 reversed the regulation of PDGF-BB on cell cycle regulators, which likely leads to the G0/G1 accumulation of HPASMCs.

### 3.3. Resveratrol Plays an Antiremodeling Effect in Rat Pulmonary Arteries

**Figure 3:**
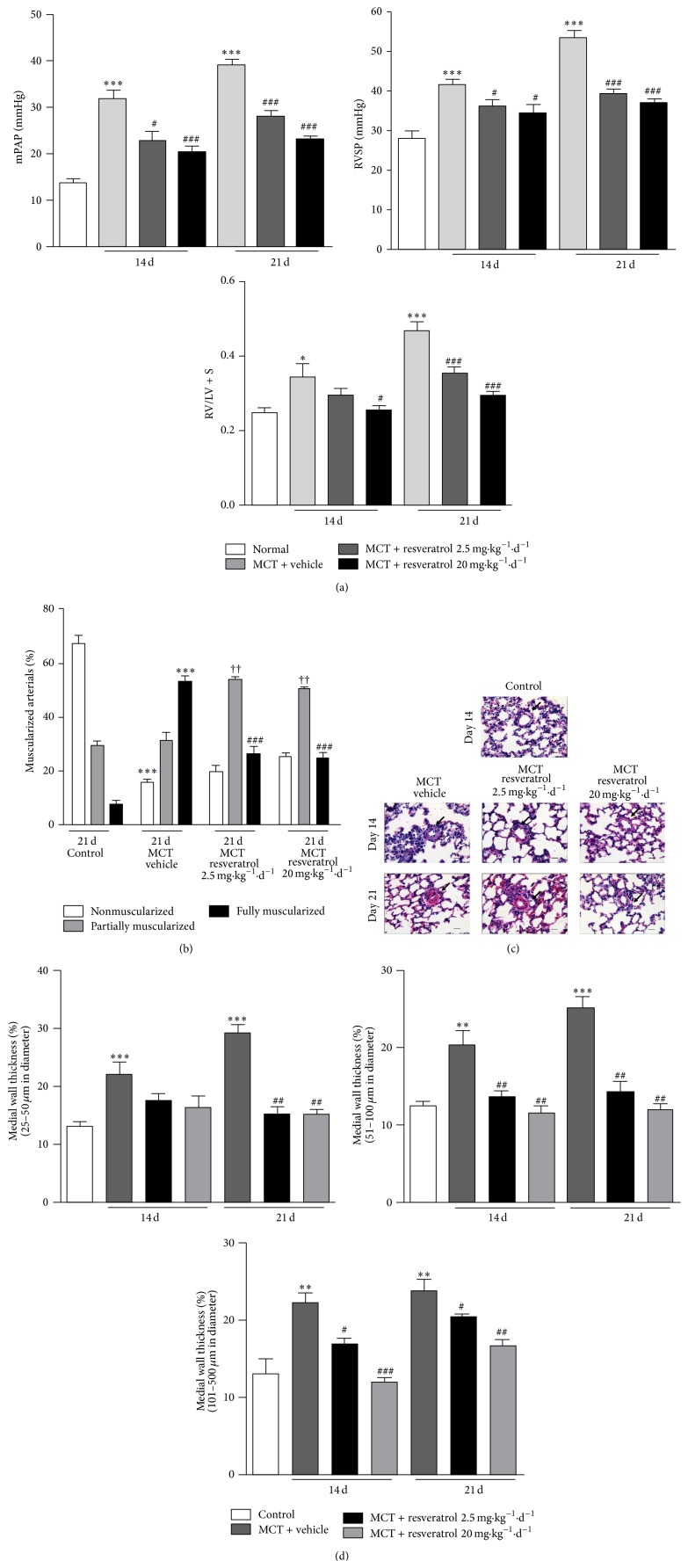
Resveratrol plays an antiremodeling effect in pulmonary arteries. (a) Hemodynamics examinations (*n* = 8–10). ^*^ *P* < 0.05, ^**^ *P* < 0.01, and ^***^ *P* < 0.001 versus normal control; ^##^ *P* < 0.01, ^###^ *P* < 0.001 versus vehicle treatment group. (b) Pulmonary arteries muscularization degrees (*n* = 5-6). ^*^ *P* < 0.05, ^***^ *P* < 0.001 versus control; ^###^ *P* < 0.001 and ^†††^ *P* < 0.001 versus corresponding vehicle group. (c) Representative H&E staining photomicrographs. (d) Pulmonary arteries medial wall thickness (*n* = 5-6). ^*^ *P* < 0.05, ^**^ *P* < 0.01, and ^***^ *P* < 0.001 versus control; ^#^ *P* < 0.05, ^##^ *P* < 0.01, and ^###^ *P* < 0.001 versus vehicle treatment group.

To elucidate the role of SIRT1 in pulmonary arterial remodeling, we constructed MCT induced PAH rat model. Real-time PCR and western blotting showed that the mRNA and protein levels of SIRT1 in the lungs of PAH rats declined dramatically 14 and 21 days after MCT treatment (Figures S3A, S3B). We chose resveratrol, the SIRT1 activator, to intervene in the PAH rat model and observed the role of SIRT1 in pulmonary arterial remodeling. All the rats treated with MCT developed PAH within 14 days. Consequently, RVSP and mPAP levels increased significantly compared with the saline-treated group. Resveratrol significantly prevented mPAP from increasing in both the 2.5 mg·kg^−1^d^−1^ group and 20 mg·kg^−1^d^−1^ groups at both 14 and 21 days (Figure 3(a)). SAP was comparable in each group (Figure S3C). As a consequence of high pulmonary arterial pressure, in the MCT-treated group, the RVHI increased 14 and 21 days after MCT challenge. Addition of resveratrol decreased RVHI in a dose-dependent manner (Figure 3(a)). To examine whether a reduction in remodeling of pulmonary arteries contributed to the amelioration of PAH rats, the degree of muscularization and the medial wall thickness of pulmonary arteries were assessed by *α*-SMA and EVG staining, respectively. The muscularization degree of pulmonary arteries with a diameter between 25 and 50 *μ*m was calculated. On day 21, with respect to controls, the percentages of both partially muscular and fully muscular pulmonary arteries increased significantly, but that of nonmuscular pulmonary arteries decreased significantly in the MCT-treated group. Resveratrol-treated groups had a markedly lower percentage of fully muscular pulmonary arteries but a higher percentage of partially muscular pulmonary arteries compared with MCT-treated vehicle treatment group (Figure 3(b) and Figure S3D). This result suggested that resveratrol attenuated the muscularization of intra-acinar arteries. Two different doses of resveratrol had comparable effects on muscularization of intra-acinar arteries (data not shown). Medial wall thickness of pulmonary arteries of diameter 25–50 *μ*m, 51–100 *μ*m, and 101–500 *μ*m was significantly increased in the MCT-treated vehicle treatment groups, both on day 14 and on day 21. Both 2.5 mg·kg^−1^d^−1^ and 20 mg·kg^−1^d^−1^ resveratrol attenuated the increase of medial wall thickness (Figures 3(c)-3(d) and Figure S3E). Treatment with resveratrol at 2.5 mg·kg^−1^d^−1^ had the comparable antithickening effect as resveratrol at 20 mg·kg^−1^d^−1^ in pulmonary arteries sized 25–50 *μ*m and 51–100 *μ*m. However, in pulmonary arteries sized 101–500 *μ*m, resveratrol at 20 mg·kg^−1^d^−1^ had a more powerful antithickening effect than resveratrol at 2.5 mg·kg^−1^d^−1^ (data not shown). No apoptosis of PASMCs was observed in any group (Figure S3F).

### 3.4. Resveratrol Increases SIRT1 and p21 Expression but Decreases Cyclin D1 Expression in Lungs of MCT-Induced PAH Rats

**Figure 4:**
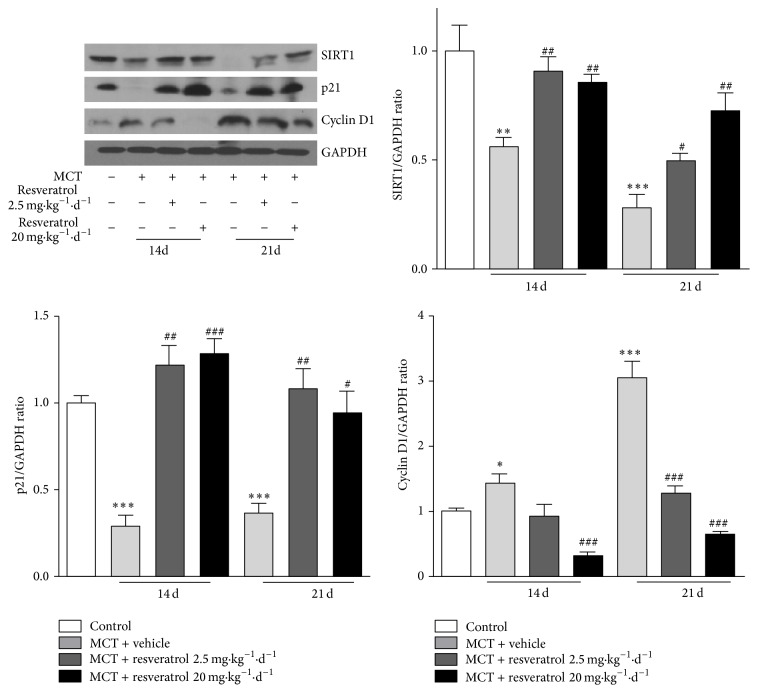
Resveratrol increases SIRT1 and p21 protein levels in PAH rats (*n* = 5). Bar graphs show densitometric analysis of western blotting. The densitometric quantification was normalized to GAPDH. Data are shown as means ± SEM for five independent experiments. ^*^ *P* < 0.05, ^**^ *P* < 0.01, and ^***^ *P* < 0.001 versus control; ^#^ *P* < 0.05, ^##^ *P* < 0.01, and ^###^ *P* < 0.001 versus vehicle treatment group on day 14 or 21.

To investigate the mechanism of resveratrol on antagonizing pulmonary arterial remodeling, the expressions of SIRT1, p21, cyclin D1, and cyclin E were detected in lungs of MCT-induced PAH rats. On both day 14 and day 21, resveratrol treatment at doses of both 2.5 mg·kg^−1^d^−1^ and 20 mg·kg^−1^d^−1^ markedly increased SIRT1 expression as compared with vehicle treatment group. p21 expression decreased significantly compared with untreated controls; however, resveratrol increased its expression markedly. Cyclin D1 expression increased significantly compared with untreated controls (Figure 4); however, resveratrol decreased its expression dramatically. No significant change in cyclin E expression was observed upon MCT challenging and resveratrol treatment did not influence cyclin E expression either (Figure S4).

### 3.5. Resveratrol Regulates the Expression of Cell Cycle Regulatory Molecules through SIRT1

**Figure 5:**
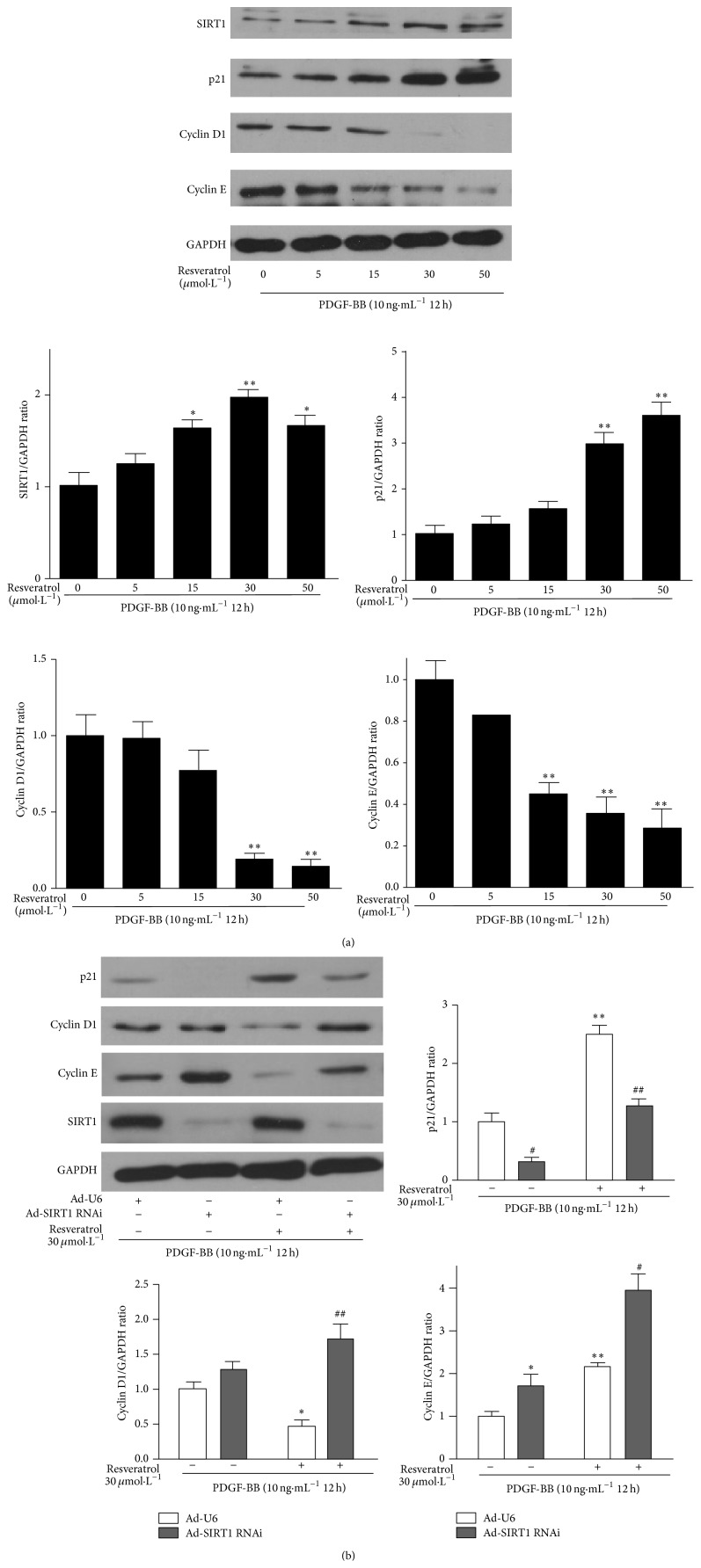
Resveratrol regulates the expression of cell cycle regulatory molecules through SIRT1. (a) Resveratrol increased SIRT1 and p21 expression but decreased cyclin D1 and cyclin E expression in HPASMCs (*n* = 3). The densitometric quantification was normalized to GAPDH. Data are shown as means ± SEM for three independent experiments. ^*^ *P* < 0.05, ^**^ *P* < 0.01 versus vehicle. (b) SIRT1 RNAi reversed the regulation of p21, cyclin D1, and cyclin E expression level by resveratrol (*n* = 3). ^*^ *P* < 0.05, ^**^ *P* < 0.01 versus vehicle pretreated Ad-U6 group; ^#^ *P* < 0.05, ^##^ *P* < 0.01 versus resveratrol pretreated Ad-U6 group.

We found that resveratrol increased SIRT1 and p21 expression but decreased cyclin D1 and cyclin E expression in a concentration-dependent manner in HPASMCs (Figure 5(a)). The activity of SIRT1 correlates positively with its level of expression [36–38]. To further investigate whether SIRT1 was required for the regulatory effect of resveratrol onthe expression of cell cycle regulatory molecules, SIRT1 was knocked down by RNA interference (RNAi) before resveratrol treatment. We found that resveratrol increased p21 expression but decreased cyclin D1 and cyclin E expressions after PDGF-BB stimulation. However, SIRT1 knockdown eliminated the effects of resveratrol on p21, cyclin D1 and cyclin E expressions (Figure 5(b)). These results suggested that resveratrol regulated p21, cyclin D1, and cyclin E expressions through SIRT1 in PDGF-BB treated HPASMCs.

## 4. Discussion

In the present study, we observed the critical role of SIRT1 in the prevention of PAH by resveratrol and investigated the underlying mechanism. There are several major findings in this study. First, SIRT1 regulated the expression of cell cycle regulators such as p21, cyclin D1, and cyclin E and arrested HPASMCs in G0/G1 phase after PDGF-BB treatment. Second, resveratrol preserved SIRT1 and p21 expressions but decreased cyclin D1 expression and exhibited an antipulmonary arterial remodeling effect on MCT induced PAH rats. This protective effect was observed at both 2.5 mg·kg^−1^d^−1^ and 20 mg·kg^−1^d^−1^ doses. Third, resveratrol regulated p21, cyclin D1, and cyclin E expressions in a SIRT1-dependent manner in PDGF-BB induced HPASMCs.

Resveratrol was reported to be a SIRT1 activator and has been shown to protect against type 2 diabetes, cancer, heart disease, inflammation, and neurodegenerative diseases [39]. Resveratrol induces apoptosis MH7A human rheumatoid arthritis synovial cells in a sirtuin 1-dependent manner. Recently, Csiszar et al. have demonstrated that resveratrol prevented monocrotaline-induced pulmonary hypertension in rats [15]. Furthermore, resveratrol significantly inhibited PDGF-BB stimulated proliferation and cellular hypertrophy in HPASMCs [40]. However, our study focused on whether SIRT1 was involved in resveratrol-mediated prevention of pulmonary arterial hypertension and the underlying mechanism. We found that SIRT1 knockdown significantly eliminated the effects of resveratrol on expressions of cell regulatory molecules such as p21, cyclin D1, and cyclin E in PDGF-BB treated HPASMCs. Resveratrol preserved the expression of SIRT1 and p21 and prevented the increase of cyclin D1 expression in the lungs of MCT-induced PAH rats.* In vitro *and* in vivo* experiments suggested that SIRT1-mediated regulation of p21 and cyclin D1 expression contributed to the antipulmonary arterial remodeling effect of resveratrol. Other mechanisms such as endothelial dysfunction and activation of inflammation take part in the pathological process of PAH. A growing body of evidence has been implicated in the role of SIRT1 in protecting the endothelium from dysfunction, inhibiting inflammation and in antioxidative stress. Whether SIRT1 mediates other beneficial actions of resveratrol also requires further elucidations.

A wide range of resveratrol concentrations (ranging from ~32 nM to 100 *μ*M* in vitro *and ~100 ng·kg^−1^ to 1500 mg·kg^−1^ body weight in animals) have been used in previous studies [41]. However, the relationship between the concentration and effects of resveratrol remains unclear. Both 2.5 mg·kg^−1^d^−1^ and 20 mg·kg^−1^d^−1^ are commonly used oral doses of resveratrol in studies of cardiovascular disease. Resveratrol at 2.5 mg·kg^−1^d^−1^ has a cardioprotective effect in diabetic- and ischemia-reperfusion-induced heart damage in rats [42, 43]. Resveratrol at 20 mg·kg^−1^d^−1^ induces myocardial angiogenesis in hypercholesterolemic rats [44]. Csiszar et al. have demonstrated the effect of 25 mg·kg^−1^d^−1^ resveratrol treatment in anti-inflammatory, antioxidant, antiproliferative, and endothelial dysfunction in the rat pulmonary arteries. In our study, we compared the effects of 2.5 mg·kg^−1^d^−1^ and 20 mg·kg^−1^d^−1^ resveratrol in PAH rat model. We found that, in the aspect of pulmonary circulation hemodynamics, the high dose group had a significantly lower mPAP and RVHI compared with low dose group; however, there were no significant differences in the effect of antimedial wall thickening in pulmonary arteries sized 25–100 *μ*m in diameter. There were no significant differences in ratios of nonmuscularized or fully muscularized intra-acinar arteries between 2.5 mg·kg^−1^d^−1^ and 20 mg·kg^−1^d^−1^ doses of resveratrol. We speculated that the differences in the antiremodeling effect between these two doses are likely to be significant if resveratrol is administered over extended periods of time. In addition to pulmonary arterial remodeling, the pathological process of PAH includes endothelial dysfunction and activation of inflammation. Whether the two doses of resveratrol have similar effects in other pathological process requires further investigation.

PASMC proliferation is central to pulmonary arterial remodeling, which requires PASMCs arrested in G0 or G1 to enter the cell cycle. p21, the cell cycle inhibitor, can cause cells in G1 arrest which contributes to the inhibition of PASMC proliferation by angiotensin converting enzyme inhibitors and the preservation of p21 is essential in suppressing MCT induced PAH in rats [45]. Cyclin D1 acts as a mitogenic signal sensor and promotes G1-to-S phase progression of cell cycle [46]. Increased cyclin D1 expression and medial VSMC proliferation have been observed in rat carotid artery balloon injury model and mouse carotid artery ligation model [47]. In this study, we found that PDGF-BB decreased p21 expression but increased cyclin D1 expression in HPASMCs; however, resveratrol reversed the effects of PDGF-BB. In the lungs of MCT induced PAH rats, p21 expression was maintained and the increase in cyclin D1 expression level was prevented by resveratrol treatment. Therefore, the antiremodeling effect of resveratrol may at least partially due to the regulation of p21 and cyclin D1 expressions. We observed that resveratrol prevented the increase of cyclin E expression after PDGF-BB treatment; however, cyclin E expression did not change in our PAH rat model. The exact role of cyclin E in proliferation of PASMCs and pulmonary arterial remodeling remains unclear. Our results suggest that the role of cyclin E in pulmonary arterial remodeling is not as important as cyclin D1 and p21.

In conclusion, our findings demonstrated that resveratrol upregulated the expression of the SIRT1 expression in PDGF-BB treated HPASMCs. Furthermore, SIRT1 mediated the role of resveratrol in regulating expression of cell cycle regulatory molecules and arresting PDGF-BB treated HPASMCs in G0/G1 phase, which contributed to the attenuation of pulmonary arterial remodeling and the alleviation of pulmonary arterial hypertension in MCT-treated rats. It suggests that SIRT1 exerts a protective role in PAH associated with rheumatic diseases and can be a potential treatment target.

## Supplementary Material

Expression of CDK2 and CDK4 have no remarkable change in PDGF-BB treated HPASMCs. Overexpression of SIRT1 decreases CDK2 protein level but not affects CDK4 expression. SIRT1 expression level and the effect of resveratrol in MCT-induced PAH rats. Expression of Cyclin E has no remarkable change in lungs of PAH rats.

## Nonstandard Abbreviations and Acronyms

PAH: Pulmonary arterial hypertension
SSc: Systemic sclerosis
MCTD: Mixed connective tissue disease
SLE: Systemic lupus erythematodes
PASMCs: Pulmonary arterial smooth muscle cells
SIRT1: Silence Information Regulator 1
PDGF-BB: Platelet-derived growth factor BB
MCT: Monocrotaline
RV: Right ventricular
RVSP: Right ventricular systolic pressure
RVHI: Right ventricular hypertrophy index
LV: Left ventricle
mPAP: Mean pulmonary arterial pressure
SAP: Systemic arterial pressure
*α*-SMA: *α*-smooth muscle action.
