# Supplementary material for: Regulation of Cell Cycle Regulators by SIRT1 Contributes to Resveratrol-Mediated Prevention of Pulmonary Arterial Hypertension
Source: Biomed Res Int. 2015 Jul 26;2015:762349. doi: 10.1155/2015/762349 (PMC4529917; doi:10.1155/2015/762349)
Supplement: Supplementary file 1 — Expression of CDK2 and CDK4 have no remarkable change in PDGF-BB treated HPASMCs. Overexpression of SIRT1 decreases CDK2 protein level but not affects CDK4 expression. SIRT1 expression level and the effect of resveratrol in MCT-induced PAH rats. Expression of Cyclin E has no remarkable change in lungs of PAH rats. [file 762349.f1.pdf]

Supporting Information

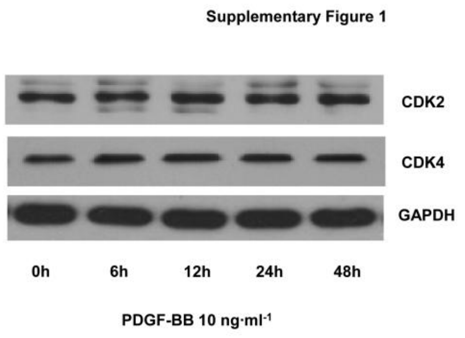

Figure S1. Expression of CDK2 and CDK4 have no remarkable change in PDGF-BB treated HPASMCs.

After 24 hours serum starvation, HPASMCs were treated with 10 ng·ml^{-1} PDGF-BB and harvested at the indicated time points. CDK2 and CDK4 protein levels were analyzed by western blotting.

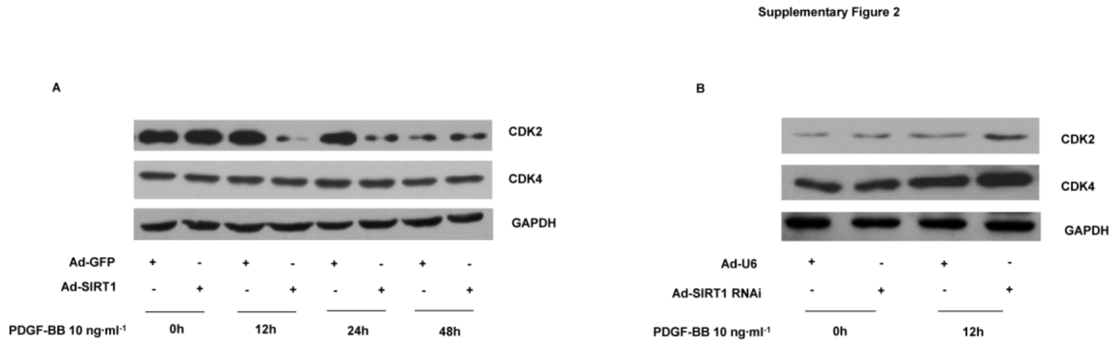

Figure S2. Overexpression of SIRT1 decreases CDK2 protein level but not affects CDK4 expression.

HPASMCs infected with Ad-SIRT1(A) or control Ad-GFP, and Ad-SIRT1 RNAi (B) or control Ad-U6 were treated with 10 $ng{\cdot}ml^{-1}$ PDGF-BB for the indicated period. CDK2 and CDK4 protein expression level were analyzed by western blotting.

Supplementary Figure 3

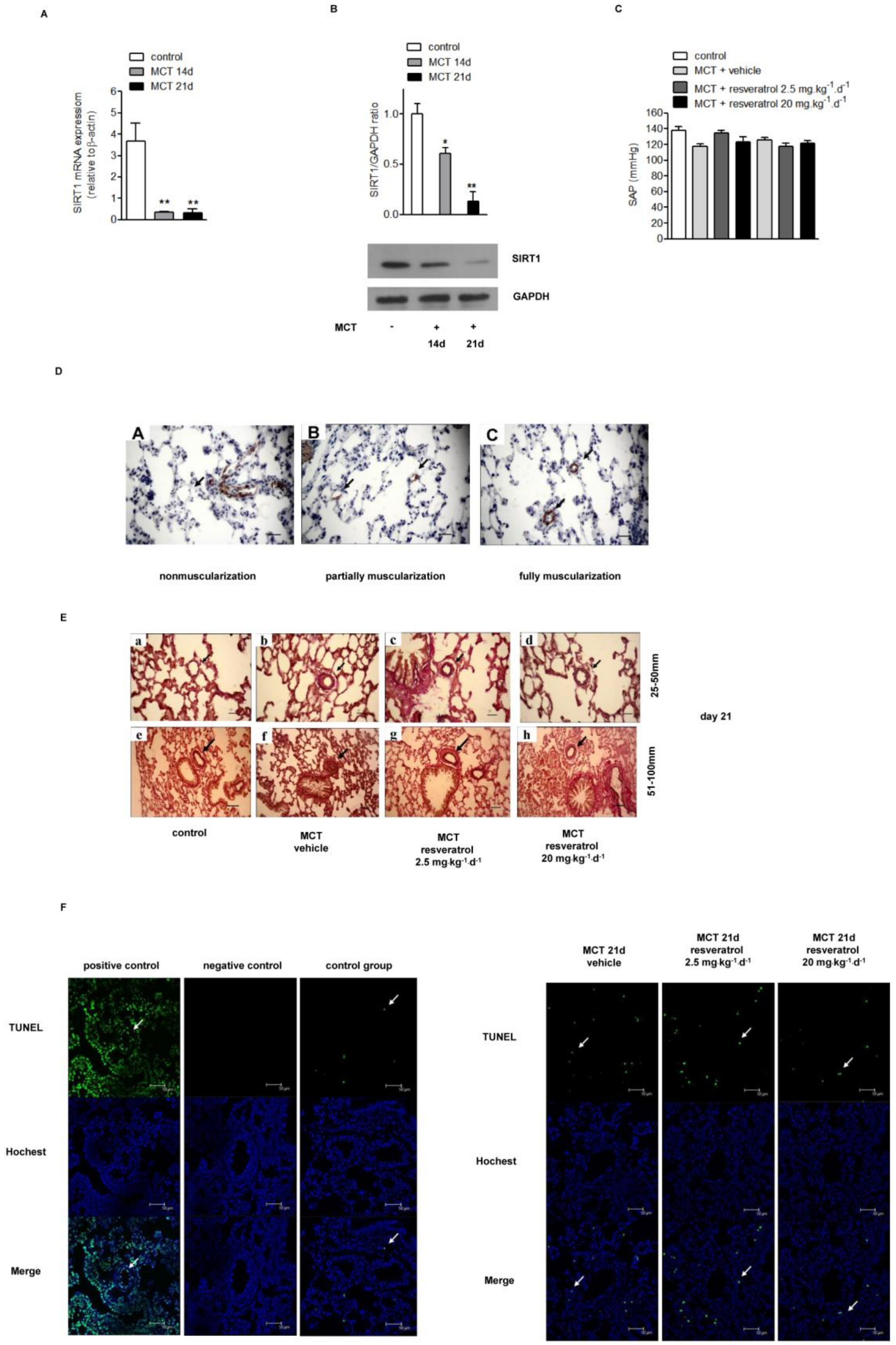

Figure S3. SIRT1 expression level and the effect of resveratrol in MCT-induced PAH rats.

(A) SIRT1 mRNA level in lungs of MCT-induced PAH rats was analyzed by real-time PCR (n=3). (B) SIRT1 protein level in lungs of MCT-induced PAH rats was analyzed by western blotting. $^*P < 0.05$, $^{**}P < 0.01$ versus control. (C) Systemic arterial pressure (SAP; in mmHg) in the different treatment group is shown. Data shown are Mean ± SEM (n=8-10). (D) Representative α-SMA staining photomicrographs of pulmonary vessels. Scale bar=25μm. (E) Representative EVG staining photomicrographs. (F) Effect of resveratrol on PASMCs apoptosis. Representative TUNEL staining photomicrographs of pulmonary vessels from each group. Scale bar=50μm.

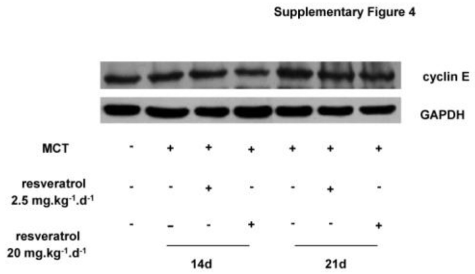

Figure S4. Expression of Cyclin E has no remarkable change in lungs of PAH rats.

Cyclin E protein level in lungs of PAH rats was detected by western blotting.
